# Impact of a Dietary Fish Oil Supplementation on the Plasma Lipidome of Healthy Adult Cats

**DOI:** 10.3390/metabo16060427

**Published:** 2026-06-18

**Authors:** Nadine Paßlack, Helena Veit, Henri Funk, Jürgen Zentek, Sven Schuchardt

**Affiliations:** 1 Small Animal Clinic, Faculty of Veterinary Medicine, Justus-Liebig-University Giessen, 35392 Giessen, Germany; 2 Statistical Consulting Unit, Ludwig-Maximilians-Universität München, 80539 Munich, Germany; helena.veit@stablab.stat.uni-muenchen.de (H.V.); henri.funk@stat.uni-muenchen.de (H.F.); 3 Institute of Animal Nutrition, Department of Veterinary Medicine, Freie Universität Berlin, 14195 Berlin, Germany; juergen.zentek@fu-berlin.de; 4 Fraunhofer Institute for Toxicology and Experimental Medicine ITEM, 30625 Hanover, Germany; sven.schuchardt@item.fraunhofer.de

**Keywords:** feline, blood, triacylglycerols, unsaturation, eicosapentaenoic acid, docosahexaenoic acid, lipidomics

## Abstract

**Background/Objectives**: Dietary fish oil supplementation has been associated with lower total plasma triacylglycerols in felines. The present study aimed to characterize this effect in more detail, using lipidomic analyses. **Methods**: Plasma samples of cats (*n* = 10), receiving a complete basic diet, with and without the addition of 0.5 g and 1.0 g fish oil/kg body weight/day, each for 21 days, in a randomized crossover design, were analyzed by an FIA MS/MS-based targeted metabolomics approach. **Results**: The results demonstrated that 360 metabolites were affected by the dietary treatments, predominantly belonging to triacylglycerols (*n* = 124), phosphatidylcholines (*n* = 68), phosphatidylethanolamines (*n* = 63), phosphatidylglycerols (*n* = 33), and phosphatidylinositols (*n* = 21). Lowering effects of fish oil supplementation on plasma triacylglycerols could be confirmed. However, increased levels of specific triacylglycerols were also observed, especially of those containing eicosapentaenoic or docosahexaenoic acid. The decreased triacylglycerols showed a lower number of carbons and a lower degree of unsaturation than the enhanced triacylglycerols. Such a lipid profile is assumed to be beneficial in human medicine; its relevance for feline health, however, is unclear so far. **Conclusions**: In conclusion, the lipidomic analyses provided a detailed characterization of the feline plasma lipidome and its modulation by a dietary fish oil supplementation. The clinical relevance of these findings warrants further investigation.

## 1. Introduction

The dietary effects of n-3 fatty acids have been of scientific and medical interest for many decades. It is well known that n-3 fatty acids are essential for a variety of biological processes, such as cell membrane composition, cell signaling, gene expression, and immune function [1,2]. Consequently, their dietary supplementation, particularly of eicosapentaenoic acid (EPA) and docosahexaenoic acid (DHA), is intensively investigated to explore potential beneficial effects in healthy and diseased organisms. Examples of dietetic applications in human medicine comprise cardiovascular, orthopedic, hepatic, neurological diseases, or cancer [2,3,4]. In veterinary medicine, particular attention has been paid to the positive impact of n-3 fatty acids in osteoarthritis in companion animals. However, additional fields of application have also been investigated, including dermatological, renal, cardiac, neurological, and gastrointestinal diseases [5]. Moreover, dogs with lipid disorders, especially hyperlipidemia, could benefit from n-3 fatty acid dietary supplementation [6,7,8].

We recently demonstrated that the intake of n-3 fatty acid-rich fish oil lowered the triacylglycerol and increased the total and low-density lipoprotein cholesterol concentrations in the plasma of healthy cats [9]. Notably, the changes in blood lipids could not be observed when sunflower oil (as a source of n-6 fatty acids) or lard (containing saturated and monounsaturated fatty acids as well as arachidonic acid) were supplemented to the diet of the cats, emphasizing the specific metabolic influence of n-3 fatty acids [9].

Although measurement of triacylglycerols and cholesterol is common practice in veterinary medicine and an important diagnostic tool, it does not allow for a more detailed characterization of blood lipids. For this, modern lipidomic analyses can be used and are already well established in human medicine research [10]. In contrast, data from veterinary medicine, particularly from cats, remain limited. To our knowledge, only one study has been performed on the feline plasma lipidome to date [11]. In this investigation, fish oil, either alone or in combination with medium-chain triacylglycerols (MCT), was included in a basic diet.

In addition to the described study of Jackson and Jewell [11], a few more studies have evaluated the fatty acid pattern in the plasma of cats [12,13,14]. Due to another laboratory technique, however, the variables assessed were limited in number compared to a comprehensive lipidomics approach. Nevertheless, the authors demonstrated significant effects of diet [12,13], species [13], as well as age and neutering [14] on specific fatty acids in feline plasma samples.

Overall, the analysis of bioactive lipid species in the blood may help to better describe and understand the lipid metabolism, the impact of its modulators, and finally, the underlying mechanisms of lipid disorders. Nutritional parameters, such as the total dietary fat concentration and fatty acid composition, could play key roles as influencing factors. Based on our previous study [9], we aimed to characterize the observed effects of dietary fish oil supplementation on the blood lipids of cats in more detail. For this, lipidomic analyses were performed in the related feline plasma samples.

## 2. Materials and Methods

### 2.1. Study Design

Plasma samples from our previous investigation [9,15] were used for the lipidomic analyses. In this randomized crossover study (Table 1), 10 healthy cats (46.6 ± 14.1 months old, initial body weight (BW): 4.99 ± 0.91 kg, 7 female neutered, 3 male neutered) were assigned to 7 dietary treatments, receiving either a complete commercial low-fat basic diet (Vet-Concept “Cat Intestinal Low Fat”, Föhren, Germany; analyzed fat concentration: 9.07 g/100 g dry matter) or the basic diet supplemented with sunflower oil (Aro, MCC Trading International GmbH, Düsseldorf, Germany), fish oil (Grizzly Lachsöl, Grizzly Pet Products, Woodinville, WA, USA), or lard (private butcher in Ulrichstein Feldkrücken, Germany). The oils and lard were added to the individual daily meal of the cats at a concentration of 0.5 g or 1.0 g per kg BW. Each feeding period lasted for 21 days, with no wash-out period in between. On day 20 of each feeding period, fasting blood samples of the cats were collected via puncture of the cephalic vein. Plasma was obtained by centrifugation of the lithium-heparinized blood at 1900× *g* and 4 °C for 10 min (Heraeus Megafuge 16 R, Thermo-Fisher Scientific, Karlsruhe, Germany). The samples were stored at −80 °C before shipment to the Fraunhofer Institute for Toxicology and Experimental Medicine ITEM (Hanover, Germany) for the lipidomics analyses. As our previous data could demonstrate an effect of the dietary fish oil supplementation on selected lipid fractions in the plasma of the cats, but no impact of the sunflower oil and lard treatment was observed [9], only the plasma samples of the feeding periods with fish oil were further analyzed and compared with the samples obtained when feeding the basic diet without an additional fat supplementation (control treatment).

More details on the study design are provided elsewhere [9,15]. All procedures were approved by the ethics committee of the relevant local authority in Giessen, Germany (Regierungspräsidium Giessen, approval number G41/2022).

The analyzed nutrient composition of the basic diet and the fish oil is presented in Table 2 and Table 3.

### 2.2. Lipidomic Analyses

The lipidomic analyses in this study were performed with the Biocrates AbsoluteIDQ 500 XL kit (Biocrates Life Sciences AG, Innsbruck, Austria) to enable targeted, quantitative profiling across multiple metabolite classes, including acylcarnitines, amino acids, hexoses, glycerophospholipids, and sphingolipids. The standard protocol for lipid species detection relies on flow injection analysis-tandem mass spectrometry (FIA MS/MS); there is no chromatographic separation in this workflow; therefore, no liquid chromatography (LC) parameters or retention times are reported. Lipid/metabolite annotation and identification were conducted in MetIDQ (Biocrates Life Sciences AG, Innsbruck, Austria), using the kit’s library of predefined Multiple Reaction Monitoring (MRM) transitions; identity is defined by the kit’s library, with blanks, matrix-matched calibrators, and quality control (QC) samples incorporated as described in the manufacturer’s protocol. Internal standards are kit-provided, stable-isotope–labeled compounds, with exact identities and panel composition specified in the kit manual. Lipids are reported as lipid species defined by the kit’s nomenclature (e.g., PC 34:2, PE 38:4), reflecting total acyl chain composition but not complete fatty acid compositions for most species.

Sample preparation was performed in 96-well plates preloaded with isotopically labeled internal standards and processed according to the manufacturer’s instructions, including phenylisothiocyanate derivatization of amino acids and biogenic amines. Lipids and fatty acids were analyzed by FIA MS/MS and quantified using single-point internal calibration with isotope correction relative to the isotopically labeled internal standards. Data acquisition was performed using Analyst software (version 1.7.2; Sciex, Darmstadt, Germany), and metabolite identification and quantification were conducted in MetIDQ (version DB116-631), including automated isotopic correction and QC monitoring; results were evaluated against the kit’s acceptance criteria. For explicit MRM transitions, library version, and related identification parameters, the Biocrates kit documentation can be used. All quantified metabolites in this study are provided as a Supplementary Material.

### 2.3. Statistical Data Evaluation

The dataset was first checked for completeness. If values were missing for one metabolite, i.e., if the concentration in a sample was below the detection limit of the analyzer, half the minimum value measured for the respective variable in the other cats was used. In total, 1668 values were replaced across 215 metabolites (604 values in the basic diet group, 541 values in the 0.5 g fish oil/kg BW/day group, and 523 values in the 1.0 g fish oil/kg BW/day group). Metabolites with fewer than 9 measured values above the detection limit (*n* = 139) as well as metabolites with the same concentration in all samples (*n* = 45) were excluded from the statistical analysis (see Supplementary Material).

The data were log_10_-transformed and then statistically tested with R (version 4.4.3). For each metabolite, a linear mixed model was fitted, with dietary treatment as a fixed effect and each cat as a random intercept, using the lme function from the nlme package (version 3.1.167). The significance of the diet effect was assessed using a marginal F-test.

The resulting *p*-values were adjusted by the Benjamini–Hochberg False Discovery Rate (FDR) method to control for multiple testing. An FDR-adjusted *p*-value < 0.05 was considered a statistically significant diet effect. Non-affected plasma metabolites are presented in the Supplementary Material. Variables with a significant diet effect were further analyzed by a Tukey-type multiple comparison procedure, testing all three pairwise contrasts simultaneously via the glht function from the multcomp package [16]. Unlike classical Tukey’s Honest Significant Difference (HSD) post-hoc test, this approach accounts for the repeated measures structure of the data. The procedure controls the familywise error rate across the three pairwise comparisons within each metabolite. No additional FDR correction was applied at the post-hoc stage, as this was already addressed by the FDR correction applied to the *p*-values of the marginal F-test.

In addition to the described statistical analysis, a principal component analysis (PCA) was conducted as an exploratory analysis, using prcomp from the stats package (version 4.4.3). By this, a potential clustering of the plasma lipidome depending on the dietary treatment of the cats was visually evaluated. For the PCA analysis, the same preprocessed and log_10_-transformed data were used as for the linear mixed model. The data matrix was mean-centered but not scaled prior to PCA. To aid visual interpretation, 95% inner quantile ellipses were drawn around each dietary group mean based on the normal distribution.

Significantly increased or decreased plasma metabolites were illustrated in volcano plots, where the log_2_ fold change was plotted on the x-axis as a measure of effect size and the −log_10_-transformed *p*-value from the post-hoc test was plotted on the y-axis as a measure of statistical significance.

Data are presented in tables as means ± standard deviations (non-log-transformed metabolite concentrations). The PCA was created with log_10_-transformed values of metabolite concentrations originally expressed in µmol/L. In the volcano plots, the log_2_ fold change values are based on the non-log-transformed metabolite concentrations, while the *p*-value from the Tukey-type post-hoc test was calculated with log_10_-transformed data.

## 3. Results

### 3.1. Health Status, Feed Intake, and Body Weight of the Cats, Apparent Nutrient Digestibility

One cat developed respiratory symptoms unrelated to the feeding trial and was temporarily withdrawn from the study until recovery. Consequently, the sample size of the control group receiving the basic diet was reduced to *n* = 9. All other cats remained clinically healthy throughout the study period.

The daily feed intake and weekly body weight of the cats did not differ among groups [9]. Moreover, the apparent nutrient digestibility was also not influenced by the dietary treatments [9].

### 3.2. Principal Component Analysis

**Figure 1:**
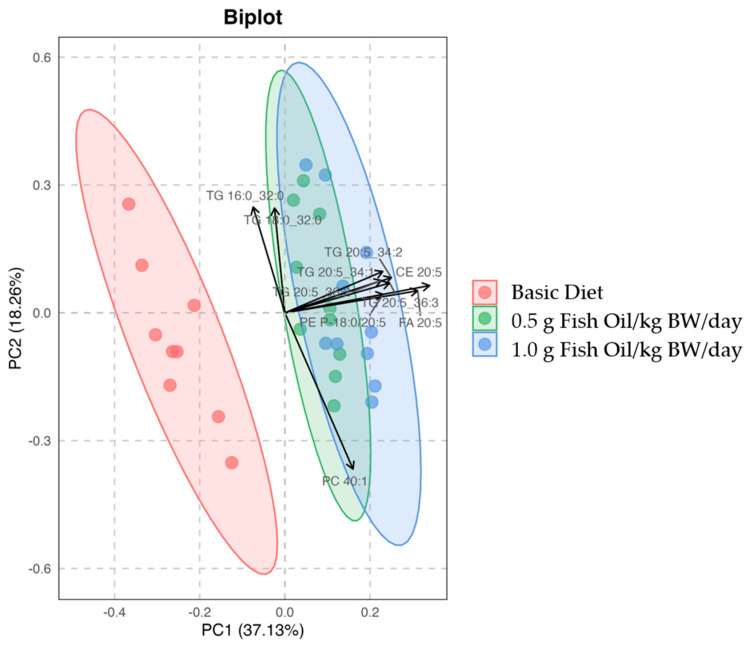
Principal component analysis of the plasma lipidome of cats fed either a basic diet without (*n* = 9) or with fish oil at a supplementation dose of 0.5 g/kg body weight (BW)/day (*n* = 10) or 1.0 g/kg BW/day (*n* = 10).

Based on the visual evaluation, the principal component analysis of the plasma lipidome revealed a clear clustering of the control group receiving the basic diet only and the two groups receiving the basic diet with the addition of fish oil (Figure 1). No marked difference in the plasma lipidome of the cats could be observed depending on the dietary supplementation dose of fish oil; however, as also indicated by the first principal component (PC1), the group receiving 1.0 g fish oil/kg BW/day showed a broader dispersion than the 0.5 g fish oil/kg BW/day group.

### 3.3. Detected Metabolites and Classification

In total, 893 metabolites, including also non-lipid species, were identified in the plasma samples of the cats. After exclusion of 139 few-positive (<9 measured values) and 45 constant variables (see Section 2.3), 709 metabolites were statistically evaluated. A significant diet effect could be detected for 360 metabolites in the plasma of the cats. These metabolites were classified into triacylglycerols (*n* = 124), phosphatidylcholines (*n* = 68), phosphatidylethanolamines (*n* = 63), phosphatidylglycerols (*n* = 33), phosphatidylinositols (*n* = 21), cholesterol esters (*n* = 11), phosphatidylserines (*n* = 8), diacylglycerols (n = 5), sphingomyelins (*n* = 5), fatty acids (n = 4), ceramides (*n* = 3), glycosylceramides (*n* = 3), phosphatidic acids (*n* = 3), and others (*n* = 9).

**Figure 2:**
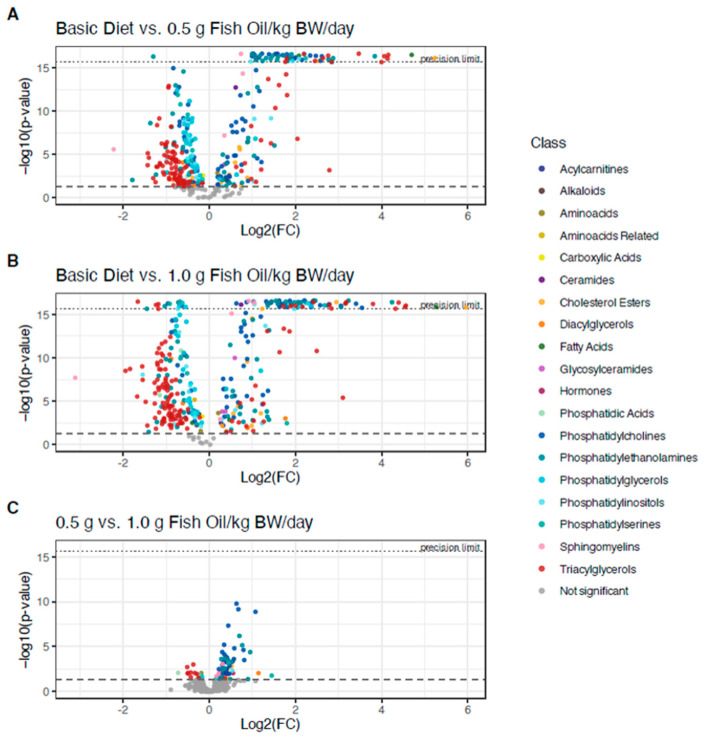
Volcano plots of pairwise post-hoc comparisons of differential plasma metabolites in cats fed a basic diet without fish oil (*n* = 9) or supplemented with fish oil at 0.5 g/kg body weight (BW)/day (*n* = 10) or 1.0 g/kg BW/day (*n* = 10). Only metabolites with a significant overall diet effect after FDR correction (FDR-adjusted *p*-value < 0.05) are displayed. The x-axis shows the log_2_ fold change (FC) (panel (**A**): Basic Diet vs. 0.5 g Fish Oil; panel (**B**): Basic Diet vs. 1.0 g Fish Oil; panel (**C**): 0.5 g vs. 1.0 g Fish Oil), and the y-axis shows the −log_10_(*p*-value) from the Tukey-type post-hoc test. Each point represents an individual metabolite, and colors indicate metabolite classes. The horizontal dashed line indicates statistical significance at a *p*-value < 0.05. If the post-hoc *p*-value of a metabolite underflowed to zero due to machine precision limits (*p* < 2.22 × 10^−16^), points are displayed above a dotted line, marking the precision limit and jittered vertically to prevent point overlap.

Changes in individual metabolites and metabolite classes are shown in the volcano plot in Figure 2.

### 3.4. Triacylglycerols

When compared to the control treatment, several plasma triacylglycerols were increased (*n* = 32) or decreased (*n* = 92) by the fish oil treatment of the cats (Table 4 and Table 5). Whilst most effects were observed independently of the supplementation dose, some triacylglycerols were especially modulated by the highest fish oil dose. Overall, the analysis of the plasma triacylglycerol concentrations showed that the reduction (373 µmol/L) induced by the dietary fish oil supplementation was substantially greater than the observed increasing effects (55.3 µmol/L).

### 3.5. Phosphatidylcholines

Most plasma phosphatidylcholines (*n* = 52) were increased in the fish oil groups when compared to the control group (Table 6), while only 16 plasma phosphatidylcholines were decreased (Table 7). Nevertheless, when the overall concentrations were considered, comparable effects were observed, with specific phosphatidylcholines increasing (537 µmol/L) or decreasing (502 µmol/L) in the comparison between the control treatment and the highest fish oil supplementation dose.

The phosphatidylcholine-lowering effect of the fish oil supplementation was observed at both doses. In contrast, the increasing effects on plasma phosphatidylcholines were more heterogeneous: 21 species were increased irrespective of the fish oil dose; 28 species were elevated in the cats receiving 0.5 g fish oil/kg BW/day compared to the control group and showed a further increase at 1.0 g fish oil/kg BW/day; and 1 species was increased only at the highest supplementation dose.

For the phosphatidylcholines PC 28:1 and PC O-40:1, group differences were noted between the control group and the group receiving 1.0 g fish oil/kg BW/day, as well as between both fish oil groups.

### 3.6. Phosphatidylethanolamines

The dietary supplementation of fish oil, independently of the dose, increased (*n* = 23) or decreased (*n* = 23) certain phosphatidylethanolamines in the plasma of the cats (Table 8 and Table 9). Moreover, some phosphatidylethanolamines were increased (LPE 18:0, LPE 22:1, PE 36:0, PE 36:6, PE 38:1, PE 38:2, PE P-18:1/20:5, PE 38:7, PE 40:1, PE 40:5, PE 40:8) by the supplementation of 0.5 g fish oil/kg BW/day when compared to the control treatment, but this effect was significantly more pronounced at the higher supplementation dose of 1 g fish oil/kg BW/day (Table 8). The plasma concentration of LPE P-18:0 and PE 34:0 was significantly higher after the dietary supplementation of 1 g fish oil per kg BW of the cats than after the control treatment or after the dietary supplementation of 0.5 g fish oil/kg BW/day. The concentration of LPE P-22:5 was the lowest in the plasma of cats receiving 0.5 g fish oil/kg BW/day. Finally, two phosphatidylethanolamines (PE 42:7, PE 42:8) were only different between the control group and the group receiving 1 g fish oil/kg BW/day, while PE 38:3 was only different between the two fish oil groups.

In general, the increasing effects of the fish oil treatment on plasma phosphatidylethanolamines (+41.0 µmol/L; comparing the control group and the group 1.0 g fish oil/kg BW/day) were more pronounced than the lowering effects (−12.3 µmol/L). It was also notable that the phosphatidylethanolamines showing the greatest increases often contained DHA or EPA.

### 3.7. Phosphatidylglycerols

In total, 33 phosphatidylglycerols were affected by the fish oil addition to the cats’ diet (Table 10). Most of these phosphatidylglycerols (*n* = 29) were decreased in the fish oil groups compared to the control group. The effects were largely independent of the supplementation dose.

### 3.8. Further Metabolites

Table 11 summarizes further plasma metabolites that differed significantly depending on the dietary treatment of the cats.

Most of the plasma phosphatidylinositols were not dose-dependently affected by the fish oil supplementation. The cholesterol esters, particularly those derived from EPA and DHA, markedly increased in the plasma when the cats received fish oil in their diet. An exception was only made by CE 20:3, which was lower, when the diets with 0.5 g and 1.0 g fish oil/kg BW/day were fed instead of the basic diet alone.

Six plasma phosphatidylserines were lowered when fish oil was added to the diet of the cats, while only two phosphatidylserines (PS 40:5, PS 40:8) were increased. However, when considering the total plasma concentrations, the enhanced phosphatidylserines (+1.18 µmol/L) slightly exceeded the decreased ones (−1.13 µmol/L).

Only five plasma diacylglycerols differed depending on the dietary treatment, while the highest fish oil supplementation dose seemed to have the strongest effect on their concentrations. Four sphingomyelin concentrations (SM 41:2, SM 42:1, SM 42:2, SM 44:2) were increased when the cats received the diets with fish oil. In contrast, SM 40:4 was decreased in these groups.

The fatty acids FA 20:5 (EPA) and FA 22:6 (DHA) were markedly (*p* < 0.001), and the fatty acid FA 20:1 (eicosenoic acid) was moderately (*p* = 0.015) increased in the plasma of the cats by the dietary fish oil supplementation, but without an additional dose effect. The plasma concentration of eicosadienoic acid (FA 20:2) was decreased by the highest dietary fish oil dose when compared to the control treatment.

The plasma concentrations of the affected ceramides (*n* = 3), glycosylceramides (*n* = 3), and phosphatidic acids (*n* = 3) were low in general. While all glycosylceramides (Hex2Cer d18:1/16:0, Hex-Cer d18:1/18:0, Hex-Cer d18:1/24:1) and the ceramide Cer d18:1/24:1 were increased by the fish oil treatment of the cats, the phosphatidic acids (PA 18:0_18:1, PA 18:0_18:2, PA 18:2_20:0) and ceramides Cer d18:1/20:0 and Cer d18:1/23:0 were decreased.

The dietary fish oil supplementation increased the concentrations of the acylcarnitines C0 and C4 in the plasma of the cats. Moreover, some non-lipid species were either increased (abscisic acid, glutamine, homoarginine) or decreased (betaine, hydroxyglutaric acid, trigonelline, valine) in the plasma of the cats by the dietary fish oil treatment.

## 4. Discussion

Lipidomic analyses allow for a more precise evaluation of blood lipids than standard procedures in routine veterinary practice. Based on the analytical technology used, targeted, untargeted, or shotgun lipidomics can be performed in different fluids, tissues, or single cells [17]. By this, metabolic pathways are specifically investigated, and relevant lipid species may be identified to serve as clinical biomarkers in the future [17,18]. Although lipidomics has been primarily applied in human medicine so far, its implementation in veterinary medicine also appears feasible, but requires further research and the development of an expanded database.

In the present study, plasma samples of cats were analyzed with triple quadrupole mass spectrometry, resulting in an output of 893 identified metabolites, including also non-lipid species. After statistical evaluation, 360 metabolites were significantly different among treatment groups, predominantly belonging to triacylglycerols (*n* = 124), phosphatidylcholines (*n* = 68), and phosphatidylethanolamines (*n* = 63).

To the best of our knowledge, only one study has been performed on the effects of a dietary fish oil supplementation on the feline plasma lipidome to date [11]; therefore, opportunities for data comparison are limited. In the investigation of Jackson and Jewell [11], 656 metabolites were identified in the plasma of cats, and 456 metabolites were significantly different among the treatment groups receiving a control diet without or with fish oil and/or MCTs. Overall, fish oil and MCTs revealed oil-specific effects on the plasma lipidome, but seemed to act also synergistically, as some results were only observed when both supplements were added together to the diet of the cats [11]. When focusing on the fish oil-only group, 265 metabolites differed compared to the baseline values, which is a lower number than observed in the present study. Moreover, the types of affected metabolites widely differed between the two studies, probably due to variations in the feeding regimen. The fish oil used by Jackson and Jewell [11] contained 36.5% DHA and 5% EPA, and was added to the diet at 2.85% on a dry matter basis. The total fat concentration of the diet was 22% in dry matter [11]. In our study, the fish oil composition was remarkably different (7.3% DHA and 13.9% EPA), and also the basic diet and supplementation doses varied. We decided to use a low-fat diet (9% crude fat in dry matter) to avoid a potential baseline effect or interfering impact of the control treatment, and to specifically evaluate the graduated supplementation of fish oil at 0.5 and 1.0 g per kg BW of the cats (corresponding to approximately 12% and 15% crude fat in dietary dry matter, respectively).

Nevertheless, despite differences in the feeding regimen and divergent effects on the feline plasma lipidome, some comparable results were observed between the study by Jackson and Jewell [11] and our investigation. In particular, the concentrations of DHA and EPA increased in the plasma of the cats when fish oil was added to the diet. Moreover, some phosphatidylcholines (LPC 18:2, LPC 24:0), phosphatidylinositols (PI 18:1_20:4, PI 18:1_18:2), and phosphatidylethanolamines (LPE 16:0, LPE 18:1, LPE 18:2, LPE 20:4) were similarly affected by the fish oil supplementation in both studies.

When interpreting the dietary impact on the plasma lipidome, it should be considered that the blood samples were collected in the fasting state of the animals. It has been demonstrated that blood triacylglycerols are markedly increased in cats postprandially but return to baseline after approximately 10 h [19]. Thus, the observed effects on plasma lipids should, in general, not reflect the short-term impact of the fatty acid ingestion, but rather a modulation of the lipid metabolism of the cats.

Interestingly, the need for fasting blood samples for lipid analyses is controversially discussed in human medicine [20,21,22]; however, a recent meta-analysis concluded that fasting samples remain preferable for reliable lipid testing [23]. On the other hand, information on the kinetics of fatty acids or other lipid fractions is limited, both in human and veterinary medicine. Regarding plasma concentrations of EPA and DHA, a recent study with human subjects demonstrated a relatively long half-life of 45 ± 8 h and 66 ± 12 h, respectively [24]. Moreover, large inter-individual variabilities in the half-life of EPA and DHA in plasma samples could be observed [24]. Whether similar effects also occur in cats has not yet been investigated. Thus, it remains unclear at this point if the observed increase of DHA and EPA in the plasma samples of the present study was a postprandial effect of the fish oil supplementation or a result of metabolic changes in the feline organism. Nevertheless, it can be concluded that the continuous dietary provision of DHA and EPA enhances their plasma concentrations also in the fasting state of cats, which is in line with previous results [11,12]. Changes in plasma levels of DHA and EPA may also contribute to alterations of the immune response and metabolic processes known to be associated with the intake of these n-3 fatty acids in felines [5,25,26].

In our previous study, we could demonstrate that the dietary supplementation of fish oil reduced the plasma concentrations of triacylglycerols in healthy cats [9]. This observation is in line with previous investigations in cats [11,27], dogs [8,28], and humans [29,30,31]. Possible mechanisms discussed to mediate the triacylglycerol-lowering effects of n-3 fatty acids basically include a modulation of specific enzymes, transcription factors, and receptors, resulting in an enhanced fatty acid oxidation and a decreased fatty acid synthesis in the liver [29,31].

The present lipidomics approach revealed further insights into the effects of a dietary fish oil supplementation on the blood triacylglycerols of cats. The general lowering impact reported before [9] could be confirmed; however, increasing effects were also detected. Those enhanced triacylglycerols particularly contained EPA or DHA, an observation that has also recently been described in human medicine [32]. Interestingly, another parallelism to the study of Lu et al. [32] was noted. The authors found that triacylglycerols with a higher number of carbons (>50 carbons) and a higher degree of unsaturation (>5 double bonds) were increased, whereas triacylglycerols with 40–60 carbons and ≤5 double bonds were decreased [32]. In line, the present investigation in cats could demonstrate an average of 55 carbons (range between 52 and 56) and an average of 7 double bonds (range 1–9) in the increased plasma triacylglycerols, but only 52 carbons (range 48–56) and 3 double bonds (range 1–7) in the decreased plasma triacylglycerols. Lu et al. [32] hypothesized that this plasma lipid profile induced by fish oil supplementation might reduce the risk for cardiovascular disease events in human subjects, as indicated by other studies [33,34,35]. However, such a potential relationship is unclear in felines so far. Future research should therefore focus on the plasma lipidome analysis of healthy and diseased cats and their potential dietary modification. For this, the present study provides a valuable starting point for an increasing database.

Evaluating the physiological relevance of the present findings in future investigations should not be restricted to the observed changes in plasma triacylglycerols. Several lipid classes were affected by the fish oil treatment in this study. Considering, however, the limited data on the feline plasma lipidome and the lack of information in diseased cats, it is impossible to discuss the metabolic changes induced by the fish oil supplementation in an interpretative context at this stage. Nevertheless, the physiological functions of specific plasma lipid fractions are well characterized. Phosphatidylcholines and phosphatidylethanolamines are the most abundant phospholipids in membranes of mammalian cells; they play major roles in the regulation of lipid metabolism, lipoprotein secretion, and energy production [36]. Taking into account that many phosphatidylcholines and phosphatidylethanolamines were increased in the plasma of the cats by the fish oil supplementation in the present study, one might speculate that this effect could have potentially contributed to the enhanced plasma lipoprotein fractions observed before [9], and to a higher plasma clearance of triacylglycerols.

Phosphatidylserines are synthesized from phosphatidylcholines and phosphatidylethanolamines and can also serve as precursors for the synthesis of phosphatidylethanolamines [37]. Given the mainly increased concentrations of phosphatidylcholines and phosphatidylethanolamines in the plasma of the cats receiving the fish oil supplementation, the slight increase of phosphatidylserines in the treated cats can be considered a coherent result. Phosphatidylserines are critical components of cellular membranes and play important roles in signaling pathways, apoptosis, and hemostasis [37].

Phosphatidylglycerols are involved in inflammatory responses of the body [38] and, thus, have been demonstrated to be enhanced in the inflammatory state [39]. The decrease in plasma phosphatidylglycerols by the fish oil supplementation in the present investigation is therefore in line with the known anti-inflammatory properties of n-3 fatty acids [40].

In this study, lipidomic analyses focused on the effects of a dietary fish oil supplementation. The samples were collected in a larger trial, which also evaluated the impact of a sunflower oil and lard treatment in cats [9,15]. As the previous results, however, did not reveal changes in blood triglycerides or cholesterol in these feeding groups [9], their samples were not considered for further lipidomic analyses. Nevertheless, it is important to mention that no final conclusions can be drawn on the modulating effects of sunflower oil or lard at this point. It is possible that these dietary treatments also affected specific plasma lipid species, although routine blood values were unchanged.

The study design should be taken into consideration when interpreting the present results. A randomized crossover design was applied to exclude sequence effects of the dietary treatments as much as possible. As a limitation, however, no wash-out period was included between the feeding periods. Moreover, given the small sample size, the statistical model did not explicitly account for the feeding period, sequence, and potential carryover effects, as such modeling would substantially reduce residual degrees of freedom. Thus, although the marked differences between the control group and the fish oil groups may reliably reflect specific diet effects, careful data interpretation is recommended.

It should finally be mentioned that different sample types can be used to assess the lipid status or potential lipid biomarkers of an individual. In the present study, plasma has been analyzed, which is a typical test material for lipidomic research [17]. However, different blood lipid pools exist, and the biological half-life of fatty acids differs between plasma, erythrocytes, platelets, and mononuclear cells [41,42]. At the moment, data on the lipidome of cats is rare in general, and only one study has been performed on the feline erythrocyte membrane lipids so far [43]. In the referenced study, however, only 11 fatty acids were analyzed, limiting a direct comparison with the comprehensive dataset generated by plasma lipidomics. Nevertheless, with ongoing research, it would be interesting to compare lipidomic data also between sample types to better evaluate the lipid metabolism and its influencing factors.

## 5. Conclusions

A dietary fish oil supplementation markedly modulated the plasma lipidome of healthy adult cats, demonstrating both general and dose-dependent effects. A triacylglycerol-lowering impact of fish oil was confirmed; however, the analyses revealed a more precise picture by showing decreasing and increasing concentrations of specific triacylglycerols. In this regard, interesting parallelisms to results from human medicine were observed; the physiological and clinical relevance for feline health needs to be investigated in the future. Overall, this study provides one of the most comprehensive characterizations of the feline plasma lipidome to date and contributes valuable reference data for further investigations.

## Figures and Tables

**Table 1:** Original study design [9,15]. Ten cats received a basic diet alone (control) or with the addition of sunflower oil, fish oil, or lard ^1^ in 7 feeding periods (FP). For the present investigation on the feline plasma lipidome, only samples of the control and fish oil treatments were used.

Cat (Number)	Basic Diet Alone	Sunflower Oil 0.5 g	Fish Oil 1.0 g	Lard 1.0 g	Fish Oil 0.5 g	Sunflower Oil 1.0 g	Lard 0.5 g
1	FP 1	FP 2	FP 3	FP 4	FP 5	FP 6	FP 7
2	FP 7	FP 1	FP 2	FP 3	FP 4	FP 5	FP 6
3	FP 6	FP 7	FP 1	FP 2	FP 3	FP 4	FP 5
4	FP 5	FP 6	FP 7	FP 1	FP 2	FP 3	FP 4
5	FP 4	FP 5	FP 6	FP 7	FP 1	FP 2	FP 3
6	FP 3	FP 4	FP 5	FP 6	FP 7	FP 1	FP 2
7	FP 2	FP 3	FP 4	FP 5	FP 6	FP 7	FP 1
8	FP 1	FP 2	FP 3	FP 4	FP 5	FP 6	FP 7
9	FP 7	FP 1	FP 2	FP 3	FP 4	FP 5	FP 6
10	FP 6	FP 7	FP 1	FP 2	FP 3	FP 4	FP 5

^1^ Supplementation dose of sunflower oil, fish oil, or lard: 0.5 g and 1.0 g per kg body weight per day.

**Table 2:** Dry matter, crude nutrient, and mineral concentrations of the basic diet used in this study ^1,2^ [9,15].

Component	Analyzed
Dry matter (g/100 g)	93.7
	In g/100 g dry matter
Crude protein	35.0
Crude fat	9.07
Crude fiber	2.24
Crude ash	11.1
Calcium	1.89
Phosphorus	1.25
Sodium	0.69
Potassium	0.97
Magnesium	0.11
	In mg/100 g dry matter
Copper	2.13
Zinc	16.9
Iron	80.6
Manganese	4.90

^1^ Analyzed by an accredited external laboratory (AGROLAB LUFA GmbH, Kiel, Germany), using the official methods for feed analyses (Commission Regulation (EC) No 152/2009 III, A, M, C, I: 2009-01, H, Procedure B: 2009-01; DIN EN 15621: 2017-10). ^2^ Composition, as specified by the manufacturer, was as follows: meat and animal by-products (duck meat meal), vegetables (dried sweet potato), plant by-products (dried tapioca, cellulose), oils and fats, flaxseed, minerals, chicory as a source of inulin (0.5%).

**Table 3:** Fatty acid concentrations in the basic diet and fish oil used in the present study ^1^ [9,15].

	Basic Diet	Basic Diet	Fish Oil
	mg/kg Diet ^2^	% of Total Fatty Acids ^3^	
Analyzed			
Caprylic acid C 8:0	<50.0	<0.1	<0.1
Capric acid C 10:0	111	0.2	<0.1
Lauric acid C 12:0	466	0.7	<0.1
Myristic acid C 14:0	478	0.7	4.5
Myristoleic acid C 14:1	78.6	0.1	0.1
Pentadecanoic acid C 15:0	70.6	0.1	0.3
Palmitic acid C 16:0	13,100	19.6	13.5
Hexadecanoic acid trans-isomers C 16:1 trans	<50.0	<0.1	<0.1
Palmitoleic acid C 16:1	2310	3.4	5.7
Hexadecadienoic acid C16:2 (n-4)	<50.0	<0.1	0.7
Hexadecatrienoic acid C16:3 (n-3)	<50.0	<0.1	<0.1
Margaric acid C 17:0	178	0.3	0.4
Heptadecenoic acid C 17:1	<50.0	<0.1	<0.1
Stearic acid C 18:0	4440	6.6	2.6
Octadecenoic acid trans-isomers C 18:1 trans	236	0.4	1.2
Oleic acid C 18:1	23,500	35.1	12.3
Petroselinic acid C 18:1	<50.0	<0.1	<0.1
cis-vaccenic acid C 18:1	1220	1.8	5.5
Octadecadienoic acid trans-isomers C 18:2 trans	122	0.2	0.9
Linoleic acid C 18:2 (n-6)	15,800	23.6	0.8
Octadecatrienoic acid trans-isomers C 18:3 trans	58.8	0.1	0.2
alpha-linolenic acid C 18:3 (n-3)	2900	4.3	0.7
gamma-linolenic acid C 18:3 (n-6)	79.8	0.1	0.1
Stearidonic acid C 18:4 (n-3)			3.5
Octadecatetraenoic acid C 18:4 (n-3)	<50.0	<0.1	
Arachidic acid C 20:0	282	0.4	<0.1
Eicosenoic acid C 20:1	311	0.5	11.2
Eicosadienoic acid C 20:2 (n-6)	98.3	0.1	0.2
Eicosatrienoic acid C 20:3 (n-3)	<50.0	<0.1	<0.1
Eicosatrienoic acid C 20:3 (n-6)	94.0	0.1	<0.1
Arachidonic acid C 20:4 (n-6)	552	0.8	0.3
Eicosatetraenoic acid C20:4 (n-3)	<50.0	<0.1	0.6
Eicosapentaenoic acid C 20:5 (n-3)	<50.0	<0.1	13.9
Heneicosanoic acid C 21:0	57.6	0.1	<0.1
Behenic acid C 22:0	174	0.3	<0.1
Docosenoic acid trans-isomers C 22:1 trans	<50.0	<0.1	<0.1
Docosenoic acid C 22:1	<50.0	<0.1	0.7
Cetoleic acid C 22:1	<50.0	<0.1	10.4
Docosadienoic acid C 22:2 (n-6)	<50.0	<0.1	<0.1
Docosatrienoic acid C 22:3	<50.0	<0.1	<0.1
Docosatetraenoic acid C 22:4 (n-6)	131	0.2	0.2
Docosapentaenoic acid C 22:5 (n-3)	<50.0	<0.1	0.9
Docosapentaenoic acid C 22:5 (n-6)	<50.0	<0.1	<0.1
Docosahexaenoic acid C 22:6 (n-3)	<50.0	<0.1	7.3
Tricosanoic acid C 23:0	<50.0	<0.1	<0.1
Lignoceric acid C 24:0	110	0.2	<0.1
Nervonic acid C 24:1	<50.0	<0.1	1.1
Calculated			
Sum saturated fatty acids	19,500	29.1	21.3
Sum monounsaturated fatty acids	27,700	41.3	48.2
Total sum fatty acids	67,000		
Sum polyunsaturated fatty acids	19,800	29.6	30.3
Sum trans fatty acids	417	0.62	2.3
n-3 fatty acids	2900	4.33	26.9
n-6 fatty acids	16,800	25.1	1.6
n-9 fatty acids	23,800	35.5	25.3
n-6:n-3 fatty acids ratio	5.79:1	5.79:1	0.06:1

^1^ Analyzed by an accredited external laboratory (AGROLAB LUFA GmbH, Kiel, Germany), using the methods of the German Society for Fat Science (DGF) (C-VI 11a: 2016 (mod.) + DGF C-VI 10a: 2016 (mod.)). ^2^ Quantitative measurements of the fatty acids in the total diet. ^3^ Relative amounts of the single fatty acids, expressed as % of the total fatty acids in the diet and oil. Values reported with a “<” symbol: below the detection limit of the analyzing laboratory.

**Table 4:** Concentrations (µmol/L) of plasma triacylglycerols (TG) of cats fed either a basic diet without or with fish oil at a supplementation dose of 0.5 or 1.0 g/kg body weight (BW)/day. Only TG that were increased by the fish oil supplementation are displayed.

	Basic Diet			Fish Oil (0.5 g/kg BW/day)			Fish Oil (1.0 g/kg BW/day)		
	(*n* = 9)			(*n* = 10)			(*n* = 10)		
TG 16:0_36:6	0.22	±	0.11 ^a^	0.63	±	0.25 ^b^	0.72	±	0.29 ^b^
TG 16:0_38:1	0.46	±	0.27 ^a^	0.60	±	0.39 ^ab^	0.83	±	0.38 ^b^
TG 16:0_38:2	0.58	±	0.32 ^a^	0.77	±	0.30 ^ab^	0.93	±	0.32 ^b^
TG 20:0_34:1	0.27	±	0.16 ^a^	0.40	±	0.16 ^b^	0.63	±	0.35 ^b^
TG 20:5_34:0	0.11	±	0.00 ^a^	0.77	±	1.03 ^b^	0.96	±	1.14 ^b^
TG 16:0_38:6	0.52	±	0.33 ^a^	1.80	±	0.60 ^b^	1.94	±	0.69 ^b^
TG 20:5_34:1	0.31	±	0.19 ^a^	5.45	±	1.89 ^b^	7.21	±	3.13 ^b^
TG 22:6_32:0	0.13	±	0.00 ^a^	0.31	±	0.14 ^b^	0.32	±	0.22 ^b^
TG 16:0_38:7	0.25	±	0.07 ^a^	1.35	±	0.42 ^b^	1.76	±	0.59 ^b^
TG 18:1_36:6	0.52	±	0.21 ^a^	0.92	±	0.38 ^b^	1.09	±	0.36 ^b^
TG 20:5_34:2	0.36	±	0.37 ^a^	6.07	±	1.99 ^b^	7.64	±	2.69 ^b^
TG 22:6_32:1	0.11	±	0.00 ^a^	0.44	±	0.24 ^b^	0.61	±	0.36 ^b^
TG 16:0_40:6	0.90	±	0.36 ^a^	1.52	±	0.58 ^b^	1.28	±	0.37 ^b^
TG 18:0_38:6	0.50	±	0.30 ^a^	1.29	±	0.54 ^b^	1.27	±	0.53 ^b^
TG 22:4_34:2	0.56	±	0.25 ^a^	0.27	±	0.17 ^b^	0.24	±	0.12 ^b^
TG 22:5_34:1	1.07	±	0.34 ^a^	2.09	±	0.71 ^b^	1.74	±	0.43 ^b^
TG 16:0_40:7	0.52	±	0.31 ^a^	1.83	±	0.75 ^b^	1.90	±	0.82 ^b^
TG 18:0_38:7	0.14	±	0.00 ^a^	0.42	±	0.15 ^b^	0.44	±	0.15 ^b^
TG 18:1_38:6	0.87	±	0.25 ^a^	2.95	±	0.70 ^b^	3.47	±	1.12 ^b^
TG 18:2_38:5	1.25	±	0.34 ^a^	1.92	±	0.66 ^b^	1.80	±	0.57 ^b^
TG 20:5_36:2	0.36	±	0.23 ^a^	5.78	±	1.33 ^b^	7.24	±	2.03 ^b^
TG 22:5_34:2	0.77	±	0.31 ^a^	1.77	±	0.64 ^b^	1.53	±	0.37 ^b^
TG 22:6_34:1	0.46	±	0.18 ^a^	3.29	±	1.23 ^b^	4.18	±	2.17 ^b^
TG 16:0_40:8	0.14	±	0.06 ^a^	0.87	±	0.48 ^b^	0.96	±	0.45 ^b^
TG 18:1_38:7	0.25	±	0.17 ^a^	1.70	±	0.27 ^b^	2.25	±	0.65 ^b^
TG 18:2_38:6	0.42	±	0.17 ^a^	1.91	±	0.50 ^b^	2.23	±	0.72 ^b^
TG 20:5_36:3	0.27	±	0.21 ^a^	4.71	±	1.04 ^b^	6.26	±	2.14 ^b^
TG 22:5_34:3	0.19	±	0.08 ^a^	0.37	±	0.21 ^b^	0.39	±	0.20 ^b^
TG 22:6_34:2	0.34	±	0.17 ^a^	3.77	±	1.60 ^b^	4.74	±	2.15 ^b^
TG 18:3_38:6	0.11	±	0.03 ^a^	0.29	±	0.14 ^b^	0.34	±	0.10 ^b^
TG 20:4_36:5	0.17	±	0.10 ^a^	0.37	±	0.20 ^b^	0.35	±	0.19 ^b^
TG 22:6_34:3	0.33	±	0.00 ^a^	1.13	±	0.49 ^b^	1.46	±	0.49 ^c^

Different superscript letters in the same row indicate a significant group difference (*p* < 0.05).

**Table 5:** Concentrations (µmol/L) of plasma triacylglycerols (TG) of cats fed either a basic diet without or with fish oil at a supplementation dose of 0.5 or 1.0 g/kg body weight (BW)/day. Only TG that were decreased by the fish oil supplementation are displayed.

	Basic Diet			Fish Oil (0.5 g/kg BW/day)			Fish Oil (1.0 g/kg BW/day)		
	(*n* = 9)			(*n* = 10)			(*n* = 10)		
TG 14:0_34:1	0.57	±	0.32 ^a^	0.35	±	0.22 ^b^	0.31	±	0.16 ^b^
TG 14:0_34:2	0.73	±	0.36 ^a^	0.37	±	0.14 ^b^	0.37	±	0.21 ^b^
TG 16:0_32:2	0.76	±	0.40 ^a^	0.47	±	0.14 ^b^	0.55	±	0.18 ^ab^
TG 18:2_30:0	0.50	±	0.24 ^a^	0.38	±	0.09 ^a^	0.27	±	0.11 ^b^
TG 14:0_34:3	0.23	±	0.13 ^a^	0.13	±	0.08 ^ab^	0.10	±	0.07 ^b^
TG 16:1_32:2	0.53	±	0.20 ^a^	0.34	±	0.12 ^b^	0.34	±	0.12 ^b^
TG 18:1_30:2	0.41	±	0.17 ^a^	0.23	±	0.11 ^b^	0.21	±	0.09 ^b^
TG 18:2_30:1	0.66	±	0.25 ^a^	0.43	±	0.15 ^b^	0.35	±	0.13 ^b^
TG 16:0_33:1	0.30	±	0.25 ^a^	0.17	±	0.16 ^b^	0.16	±	0.17 ^b^
TG 18:1_31:0	1.00	±	0.14 ^a^	0.89	±	0.13 ^b^	0.85	±	0.09 ^b^
TG 16:0_33:2	0.21	±	0.16 ^a^	0.09	±	0.06 ^b^	0.09	±	0.07 ^b^
TG 14:0_36:1	3.58	±	0.95 ^a^	2.69	±	0.40 ^b^	2.55	±	0.29 ^b^
TG 16:0_34:1	6.57	±	5.34 ^a^	4.48	±	1.68 ^ab^	4.04	±	2.46 ^b^
TG 18:0_32:1	0.58	±	0.37 ^a^	0.30	±	0.24 ^b^	0.27	±	0.24 ^b^
TG 18:1_32:0	3.97	±	3.27 ^a^	2.91	±	1.14 ^a^	2.16	±	1.46 ^b^
TG 14:0_36:2	2.44	±	1.35 ^a^	1.15	±	0.55 ^b^	0.84	±	0.35 ^b^
TG 16:0_34:2	9.86	±	6.11 ^a^	6.52	±	1.87 ^b^	5.66	±	2.76 ^b^
TG 16:1_34:1	5.05	±	2.54 ^a^	2.89	±	0.46 ^b^	2.55	±	1.15 ^b^
TG 18:1_32:1	8.93	±	4.31 ^a^	4.50	±	1.32 ^b^	3.93	±	1.78 ^b^
TG 18:2_32:0	3.53	±	2.06 ^a^	2.71	±	0.83 ^ab^	2.42	±	1.33 ^b^
TG 14:0_36:3	2.70	±	1.24 ^a^	1.48	±	0.66 ^b^	1.20	±	0.53 ^b^
TG 16:0_34:3	4.75	±	2.60 ^a^	2.92	±	0.73 ^b^	2.87	±	1.35 ^b^
TG 16:1_34:2	5.18	±	2.53 ^a^	3.08	±	0.74 ^b^	2.85	±	1.14 ^b^
TG 18:1_32:2	3.80	±	1.72 ^a^	2.04	±	0.72 ^b^	1.65	±	0.69 ^b^
TG 18:2_32:1	6.01	±	2.96 ^a^	3.44	±	0.94 ^b^	3.06	±	1.39 ^b^
TG 14:0_36:4	1.26	±	0.73 ^a^	0.70	±	0.30 ^b^	0.60	±	0.21 ^b^
TG 16:1_34:3	1.70	±	0.95 ^a^	0.89	±	0.33 ^b^	0.82	±	0.31 ^b^
TG 18:1_32:3	0.53	±	0.25 ^a^	0.32	±	0.17 ^b^	0.26	±	0.11 ^b^
TG 18:2_32:2	2.31	±	1.09 ^a^	1.37	±	0.48 ^b^	1.23	±	0.47 ^b^
TG 16:0_35:2	0.83	±	0.51 ^a^	0.49	±	0.14 ^a^	0.35	±	0.29 ^b^
TG 17:0_34:2	0.74	±	0.36 ^a^	0.47	±	0.12 ^ab^	0.42	±	0.22 ^b^
TG 17:1_34:1	0.54	±	0.34 ^a^	0.22	±	0.19 ^b^	0.23	±	0.18 ^b^
TG 18:1_33:1	2.13	±	1.07 ^a^	0.92	±	0.33 ^b^	0.92	±	0.57 ^b^
TG 16:0_35:3	0.47	±	0.25 ^a^	0.23	±	0.10 ^b^	0.20	±	0.12 ^b^
TG 17:1_34:2	0.52	±	0.25 ^a^	0.26	±	0.13 ^b^	0.26	±	0.14 ^b^
TG 18:1_33:2	1.27	±	0.65 ^a^	0.68	±	0.20 ^b^	0.60	±	0.29 ^b^
TG 18:2_33:1	1.73	±	0.84 ^a^	0.86	±	0.25 ^b^	0.76	±	0.30 ^b^
TG 18:1_33:3	0.22	±	0.11 ^a^	0.10	±	0.03 ^b^	0.12	±	0.04 ^b^
TG 18:2_33:2	0.79	±	0.39 ^a^	0.46	±	0.13 ^b^	0.44	±	0.17 ^b^
TG 16:0_36:2	43.2	±	22.5 ^a^	25.0	±	6.82 ^b^	21.3	±	11.1 ^b^
TG 16:1_36:1	2.07	±	0.96 ^a^	1.53	±	0.34 ^ab^	1.32	±	0.63 ^b^
TG 18:0_34:2	4.43	±	2.46 ^a^	3.22	±	0.87 ^ab^	2.82	±	1.35 ^b^
TG 18:1_34:1	66.1	±	30.0 ^a^	36.3	±	9.31 ^b^	31.4	±	17.6 ^b^
TG 16:0_36:3	59.4	±	31.2 ^a^	37.0	±	10.9 ^b^	31.7	±	15.7 ^b^
TG 16:1_36:2	9.60	±	3.70 ^a^	4.79	±	1.22 ^b^	4.16	±	1.44 ^b^
TG 18:0_34:3	1.39	±	0.61 ^a^	0.98	±	0.24 ^ab^	0.87	±	0.29 ^b^
TG 18:1_34:2	65.4	±	32.2 ^a^	38.2	±	9.52 ^b^	33.7	±	15.7 ^b^
TG 18:2_34:1	51.3	±	24.3 ^a^	32.0	±	8.41 ^b^	28.0	±	13.7 ^b^
TG 16:0_36:4	25.3	±	12.5 ^a^	17.0	±	5.80 ^b^	15.0	±	7.05 ^b^
TG 16:1_36:3	11.8	±	5.17 ^a^	6.02	±	1.50 ^b^	5.47	±	1.80 ^b^
TG 18:1_34:3	14.6	±	6.30 ^a^	7.74	±	1.91 ^b^	7.12	±	2.48 ^b^
TG 18:2_34:2	42.9	±	22.5 ^a^	28.8	±	9.95 ^b^	25.2	±	12.0 ^b^
TG 18:3_34:1	5.69	±	2.65 ^a^	3.37	±	0.98 ^b^	2.91	±	1.34 ^b^
TG 16:1_36:4	5.01	±	2.41 ^a^	2.87	±	0.76 ^b^	2.45	±	0.78 ^b^
TG 18:1_34:4	1.42	±	0.55 ^a^	1.00	±	0.29 ^b^	0.99	±	0.32 ^b^
TG 18:2_34:3	9.07	±	4.56 ^a^	5.50	±	1.50 ^b^	4.71	±	1.87 ^b^
TG 18:3_34:2	4.46	±	2.03 ^a^	2.90	±	0.83 ^b^	2.41	±	0.99 ^b^
TG 18:3_34:3	1.08	±	0.49 ^a^	0.68	±	0.32 ^b^	0.58	±	0.22 ^b^
TG 17:0_36:3	2.96	±	1.23 ^a^	1.70	±	0.52 ^b^	1.37	±	0.56 ^b^
TG 18:1_35:2	2.72	±	1.12 ^a^	1.53	±	0.49 ^b^	1.25	±	0.49 ^b^
TG 18:2_35:1	2.86	±	1.00 ^a^	1.85	±	0.61 ^b^	1.51	±	0.51 ^b^
TG 17:0_36:4	1.29	±	0.56 ^a^	0.86	±	0.22 ^b^	0.71	±	0.27 ^b^
TG 17:1_36:3	0.99	±	0.33 ^a^	0.46	±	0.16 ^b^	0.44	±	0.23 ^b^
TG 18:1_35:3	0.96	±	0.38 ^a^	0.54	±	0.12 ^b^	0.44	±	0.19 ^b^
TG 18:2_35:2	1.82	±	0.74 ^a^	1.12	±	0.29 ^b^	1.00	±	0.42 ^b^
TG 17:1_36:4	0.41	±	0.24 ^a^	0.27	±	0.13 ^ab^	0.20	±	0.11 ^b^
TG 18:2_35:3	0.62	±	0.23 ^a^	0.36	±	0.12 ^b^	0.31	±	0.13 ^b^
TG 18:3_35:2	0.32	±	0.17 ^a^	0.13	±	0.13 ^b^	0.12	±	0.10 ^b^
TG 18:0_36:2	8.19	±	4.09 ^a^	5.39	±	1.64 ^b^	4.26	±	1.75 ^b^
TG 18:0_36:3	11.3	±	4.13 ^a^	7.81	±	1.96 ^b^	6.04	±	2.74 ^c^
TG 18:1_36:2	42.6	±	15.4 ^a^	22.1	±	6.10 ^b^	18.1	±	8.15 ^c^
TG 18:2_36:1	10.8	±	4.20 ^a^	7.96	±	1.85 ^b^	6.30	±	2.78 ^c^
TG 20:2_34:1	0.78	±	0.34 ^a^	0.30	±	0.15 ^b^	0.20	±	0.12 ^b^
TG 16:0_38:4	1.37	±	0.51 ^a^	0.74	±	0.22 ^b^	0.60	±	0.24 ^b^
TG 18:0_36:4	5.32	±	1.77 ^a^	4.10	±	1.18 ^ab^	3.29	±	1.46 ^b^
TG 18:1_36:3	46.6	±	18.0 ^a^	24.3	±	7.03 ^b^	20.6	±	9.14 ^c^
TG 18:2_36:2	29.2	±	10.9 ^a^	17.2	±	5.08 ^b^	14.4	±	6.60 ^b^
TG 18:3_36:1	1.53	±	0.57 ^a^	1.10	±	0.30 ^b^	0.97	±	0.47 ^b^
TG 20:2_34:2	0.89	±	0.36 ^a^	0.33	±	0.14 ^b^	0.25	±	0.13 ^b^
TG 20:3_34:1	0.99	±	0.43 ^a^	0.44	±	0.21 ^b^	0.31	±	0.20 ^c^
TG 16:1_38:4	0.32	±	0.18 ^a^	0.12	±	0.05 ^b^	0.15	±	0.08 ^b^
TG 18:1_36:4	20.3	±	7.43 ^a^	11.5	±	3.44 ^b^	9.48	±	3.63 ^b^
TG 18:2_36:3	23.5	±	9.45 ^a^	13.7	±	5.09 ^b^	11.7	±	5.44 ^b^
TG 18:3_36:2	5.21	±	1.77 ^a^	2.92	±	0.82 ^b^	2.32	±	0.93 ^b^
TG 20:3_34:2	0.87	±	0.39 ^a^	0.39	±	0.24 ^b^	0.31	±	0.17 ^b^
TG 20:4_34:1	2.50	±	1.07 ^a^	2.33	±	0.76 ^a^	1.73	±	0.58 ^b^
TG 18:2_36:4	10.7	±	4.46 ^a^	6.69	±	2.46 ^b^	5.64	±	2.44 ^b^
TG 18:3_36:3	5.71	±	2.07 ^a^	3.30	±	1.11 ^b^	2.72	±	0.88 ^b^
TG 18:3_36:4	2.20	±	0.88 ^a^	1.47	±	0.72 ^b^	1.16	±	0.31 ^b^
TG 18:2_38:4	1.73	±	0.52 ^a^	1.16	±	0.43 ^b^	1.04	±	0.47 ^b^
TG 20:3_36:3	1.00	±	0.36 ^a^	0.45	±	0.14 ^b^	0.34	±	0.20 ^b^
TG 20:3_36:4	0.42	±	0.23 ^a^	0.18	±	0.11 ^b^	0.13	±	0.04 ^b^

Different superscript letters in the same row indicate a significant group difference (*p* < 0.05).

**Table 6:** Concentrations (µmol/L) of plasma phosphatidylcholines (PC) of cats fed either a basic diet without or with fish oil at a supplementation dose of 0.5 or 1.0 g/kg body weight (BW)/day. Only PC that were increased by the fish oil supplementation are displayed.

	Basic Diet			Fish Oil (0.5 g/kg BW/day)			Fish Oil (1.0 g/kg BW/day)		
	(*n* = 9)			(*n* = 10)			(*n* = 10)		
LPC 18:0	78.5	±	9.84 ^a^	90.6	±	10.3 ^b^	98.1	±	8.24 ^b^
LPC 24:0	0.23	±	0.05 ^a^	0.34	±	0.09 ^b^	0.40	±	0.12 ^b^
PC 24:0	0.09	±	0.02 ^a^	0.19	±	0.10 ^b^	0.21	±	0.12 ^b^
LPC 26:0	0.10	±	0.06 ^a^	0.18	±	0.08 ^b^	0.23	±	0.06 ^b^
LPC 26:1	0.07	±	0.03 ^a^	0.16	±	0.08 ^b^	0.18	±	0.07 ^b^
PC 28:1	1.49	±	0.39 ^a^	1.64	±	0.27 ^a^	1.92	±	0.35 ^b^
PC O-28:1	0.38	±	0.09 ^a^	0.64	±	0.24 ^b^	0.69	±	0.25 ^b^
PC 30:0	2.53	±	0.67 ^a^	3.04	±	0.55 ^b^	3.59	±	0.75 ^c^
PC O-30:0	0.25	±	0.06 ^a^	0.31	±	0.05 ^b^	0.39	±	0.07 ^c^
PC O-30:2	0.11	±	0.02 ^a^	0.16	±	0.03 ^b^	0.19	±	0.04 ^b^
PC 32:0	5.90	±	1.12 ^a^	6.76	±	0.92 ^b^	7.42	±	1.04 ^b^
PC O-32:2	0.45	±	0.12 ^a^	0.52	±	0.09 ^b^	0.59	±	0.08 ^c^
PC 32:3	0.23	±	0.06 ^a^	0.27	±	0.05 ^ab^	0.30	±	0.06 ^b^
PC O-34:0	1.18	±	0.29 ^a^	2.76	±	0.57 ^b^	3.69	±	0.91 ^c^
PC 36:0	0.72	±	0.42 ^a^	2.81	±	0.96 ^b^	4.93	±	1.97 ^c^
PC O-36:0	1.22	±	0.33 ^a^	2.01	±	0.36 ^b^	2.48	±	0.52 ^c^
PC 36:5	9.78	±	3.62 ^a^	58.7	±	21.0 ^b^	81.3	±	22.5 ^c^
PC O-36:5	2.13	±	0.63 ^a^	4.32	±	0.94 ^b^	5.33	±	1.08 ^c^
PC 36:6	0.35	±	0.16 ^a^	1.54	±	0.51 ^b^	2.07	±	0.64 ^c^
PC 38:0	1.33	±	0.27 ^a^	2.94	±	0.54 ^b^	4.00	±	1.02 ^c^
PC O-38:0	2.19	±	0.70 ^a^	6.57	±	1.51 ^b^	7.57	±	1.59 ^b^
PC 38:1	1.17	±	0.30 ^a^	7.12	±	1.65 ^b^	12.3	±	4.26 ^c^
PC 38:5	41.2	±	14.3 ^a^	209	±	58.4 ^b^	277	±	89.3 ^c^
PC O-38:5	6.07	±	1.87 ^a^	12.9	±	3.00 ^b^	16.2	±	4.18 ^c^
PC 38:6	20.3	±	6.77 ^a^	60.2	±	12.4 ^b^	74.1	±	14.3 ^c^
PC O-38:6	1.64	±	0.54 ^a^	5.72	±	1.03 ^b^	7.22	±	1.63 ^c^
PC 40:1	0.52	±	0.30 ^a^	1.50	±	1.07 ^b^	2.39	±	1.77 ^c^
PC O-40:1	1.60	±	0.44 ^a^	1.79	±	0.27 ^a^	2.12	±	0.39 ^b^
PC 40:2	0.72	±	0.14 ^a^	1.52	±	0.19 ^b^	2.22	±	0.61 ^c^
PC O-40:2	1.53	±	0.34 ^a^	2.23	±	0.29 ^b^	2.55	±	0.35 ^b^
PC 40:3	0.86	±	0.17 ^a^	1.78	±	0.20 ^b^	2.32	±	0.51 ^c^
PC O-40:4	5.50	±	1.59 ^a^	7.55	±	1.46 ^b^	8.74	±	2.27 ^b^
PC 40:5	9.63	±	3.16 ^a^	15.0	±	3.28 ^b^	17.4	±	4.68 ^b^
PC O-40:5	2.36	±	0.72 ^a^	7.67	±	1.26 ^b^	10.1	±	2.48 ^c^
PC 40:6	15.4	±	6.96 ^a^	61.3	±	11.7 ^b^	76.6	±	17.8 ^b^
PC O-40:6	1.27	±	0.49 ^a^	4.84	±	0.83 ^b^	6.54	±	1.69 ^c^
PC 42:0	0.20	±	0.05 ^a^	0.27	±	0.05 ^b^	0.29	±	0.07 ^b^
PC O-42:0	0.44	±	0.07 ^a^	0.88	±	0.18 ^b^	1.36	±	0.39 ^c^
PC 42:1	0.14	±	0.03 ^a^	0.31	±	0.07 ^b^	0.43	±	0.11 ^c^
PC O-42:1	0.82	±	0.21 ^a^	1.07	±	0.21 ^b^	1.24	±	0.22 ^b^
PC 42:2	0.30	±	0.07 ^a^	0.40	±	0.05 ^b^	0.53	±	0.11 ^c^
PC O-42:2	0.52	±	0.15 ^a^	1.05	±	0.26 ^b^	1.19	±	0.35 ^b^
PC O-42:3	0.46	±	0.10 ^a^	0.79	±	0.14 ^b^	0.86	±	0.20 ^b^
PC 42:4	0.28	±	0.06 ^a^	0.42	±	0.09 ^b^	0.51	±	0.12 ^b^
PC O-42:4	0.51	±	0.14 ^a^	0.69	±	0.12 ^b^	0.84	±	0.23 ^b^
PC 42:5	0.28	±	0.07 ^a^	1.00	±	0.17 ^b^	1.49	±	0.40 ^c^
PC O-42:5	0.91	±	0.20 ^a^	2.38	±	0.36 ^b^	3.00	±	0.75 ^b^
PC 42:6	0.46	±	0.16 ^a^	2.57	±	0.70 ^b^	5.39	±	2.34 ^c^
PC O-44:3	0.14	±	0.02 ^a^	0.25	±	0.07 ^b^	0.28	±	0.08 ^b^
PC O-44:4	0.14	±	0.02 ^a^	0.18	±	0.03 ^b^	0.23	±	0.04 ^c^
PC O-44:5	0.14	±	0.03 ^a^	0.25	±	0.03 ^b^	0.36	±	0.11 ^c^
PC O-44:6	0.17	±	0.02 ^a^	0.25	±	0.04 ^b^	0.32	±	0.07 ^c^

Different superscript letters in the same row indicate a significant group difference (*p* < 0.05).

**Table 7:** Concentrations (µmol/L) of plasma phosphatidylcholines (PC) of cats fed either a basic diet without or with fish oil at a supplementation dose of 0.5 or 1.0 g/kg body weight (BW)/day. Only PC that were decreased by the fish oil supplementation are displayed.

	Basic Diet			Fish Oil (0.5 g/kg BW/day)			Fish Oil (1.0 g/kg BW/day)		
	(*n* = 9)			(*n* = 10)			(*n* = 10)		
LPC 18:2	35.9	±	7.15 ^a^	25.5	±	4.32 ^b^	24.3	±	2.17 ^b^
LPC 20:3	1.42	±	0.32 ^a^	0.83	±	0.13 ^b^	0.78	±	0.08 ^b^
PC 34:1	134	±	34.7 ^a^	92.9	±	13.7 ^b^	93.2	±	12.8 ^b^
PC 34:2	231	±	58.7 ^a^	152	±	37.0 ^b^	134	±	21.8 ^b^
PC O-34:2	12.5	±	3.45 ^a^	10.3	±	2.46 ^b^	10.0	±	1.93 ^b^
PC 34:3	17.7	±	6.38 ^a^	11.0	±	3.07 ^b^	10.6	±	2.15 ^b^
PC O-34:3	2.75	±	0.93 ^a^	2.15	±	0.61 ^b^	2.05	±	0.47 ^b^
PC 36:1	140	±	35.5 ^a^	109	±	14.1 ^b^	111	±	17.8 ^b^
PC 36:2	508	±	103 ^a^	362	±	60.6 ^b^	326	±	40.0 ^b^
PC O-36:2	24.7	±	7.42 ^a^	16.5	±	3.05 ^b^	16.0	±	2.47 ^b^
PC 36:3	117	±	33.9 ^a^	65.9	±	17.0 ^b^	60.4	±	12.3 ^b^
PC O-36:3	7.09	±	2.07 ^a^	5.49	±	1.09 ^b^	5.51	±	0.85 ^b^
PC 36:4	92.6	±	24.3 ^a^	57.3	±	11.9 ^b^	50.4	±	7.05 ^b^
PC O-38:2	13.4	±	3.95 ^a^	8.26	±	1.67 ^b^	7.87	±	1.30 ^b^
PC 38:3	45.2	±	12.6 ^a^	31.6	±	3.64 ^b^	30.3	±	2.23 ^b^
PC O-38:3	5.29	±	1.50 ^a^	3.76	±	0.55 ^b^	3.81	±	0.63 ^b^

Different superscript letters in the same row indicate a significant group difference (*p* < 0.05).

**Table 8:** Concentrations (µmol/L) of plasma phosphatidylethanolamines (PE) of cats fed either a basic diet without or with fish oil at a supplementation dose of 0.5 or 1.0 g/kg body weight (BW)/day. Only PE that were increased by the fish oil supplementation are displayed.

	Basic Diet			Fish Oil (0.5 g/kg BW/day)			Fish Oil (1.0 g/kg BW/day)		
	(*n* = 9)			(*n* = 10)			(*n* = 10)		
LPE 16:0	0.70	±	0.13 ^a^	1.31	±	0.36 ^b^	1.41	±	0.52 ^b^
LPE P-16:0	0.26	±	0.04 ^a^	0.32	±	0.03 ^b^	0.37	±	0.08 ^b^
LPE 18:0	2.27	±	0.35 ^a^	2.82	±	0.38 ^b^	3.17	±	0.59 ^c^
LPE P-18:0	0.69	±	0.07 ^a^	0.75	±	0.13 ^a^	0.92	±	0.29 ^b^
LPE 20:1	0.04	±	0.00 ^a^	0.08	±	0.03 ^b^	0.09	±	0.04 ^b^
LPE 22:1	0.03	±	0.00 ^a^	0.09	±	0.06 ^b^	0.17	±	0.09 ^c^
LPE 20:5	0.03	±	0.01 ^a^	0.23	±	0.07 ^b^	0.28	±	0.12 ^b^
LPE 22:6	0.20	±	0.07 ^a^	0.67	±	0.17 ^b^	0.79	±	0.28 ^b^
PE 32:2	0.04	±	0.02 ^a^	0.08	±	0.01 ^b^	0.08	±	0.03 ^b^
PE 34:0	0.02	±	0.00 ^a^	0.03	±	0.01 ^a^	0.05	±	0.03 ^b^
PE P-20:0/14:0	0.38	±	0.11 ^a^	0.53	±	0.13 ^b^	0.62	±	0.17 ^b^
PE 36:0	0.24	±	0.07 ^a^	0.71	±	0.16 ^b^	1.01	±	0.34 ^c^
PE 36:5	0.09	±	0.02 ^a^	0.21	±	0.04 ^b^	0.25	±	0.09 ^b^
PE P-16:0/20:5	0.10	±	0.05 ^a^	0.74	±	0.23 ^b^	0.95	±	0.25 ^b^
PE 36:6	0.03	±	0.01 ^a^	0.10	±	0.02 ^b^	0.12	±	0.04 ^c^
PE 38:0	0.37	±	0.13 ^a^	1.12	±	0.28 ^b^	1.39	±	0.42 ^b^
PE 38:1	0.34	±	0.06 ^a^	0.71	±	0.09 ^b^	1.15	±	0.51 ^c^
PE P-18:0/20:1	0.38	±	0.38 ^a^	0.50	±	0.16 ^b^	0.66	±	0.19 ^b^
PE 38:2	0.31	±	0.06 ^a^	0.67	±	0.16 ^b^	1.14	±	0.62 ^c^
PE 38:3	0.30	±	0.07 ^ab^	0.27	±	0.03 ^a^	0.32	±	0.05 ^b^
PE 38:5	0.70	±	0.23 ^a^	1.48	±	0.41 ^b^	1.79	±	0.75 ^b^
PE P-18:0/20:5	0.20	±	0.12 ^a^	2.87	±	0.77 ^b^	3.78	±	0.97 ^b^
PE 38:6	0.45	±	0.17 ^a^	1.13	±	0.25 ^b^	1.37	±	0.56 ^b^
PE P-16:0/22:6	3.23	±	0.72 ^a^	8.94	±	3.46 ^b^	9.78	±	3.24 ^b^
PE P-18:1/20:5	0.17	±	0.00 ^a^	0.47	±	0.12 ^b^	0.60	±	0.13 ^c^
PE 38:7	0.14	±	0.03 ^a^	0.28	±	0.04 ^b^	0.36	±	0.09 ^c^
PE 40:1	0.09	±	0.03 ^a^	0.16	±	0.02 ^b^	0.22	±	0.07 ^c^
PE P-18:0/22:1	0.05	±	0.02 ^a^	0.39	±	0.16 ^b^	0.49	±	0.16 ^b^
PE 40:3	0.13	±	0.05 ^a^	0.24	±	0.04 ^b^	0.28	±	0.05 ^b^
PE 40:5	0.26	±	0.08 ^a^	0.34	±	0.05 ^b^	0.45	±	0.11 ^c^
PE P-18:0/22:5	2.11	±	0.53 ^a^	3.89	±	1.31 ^b^	4.15	±	1.44 ^b^
PE 40:6	0.40	±	0.15 ^a^	1.45	±	0.33 ^b^	1.90	±	0.79 ^b^
PE P-18:0/22:6	3.87	±	1.11 ^a^	14.5	±	4.75 ^b^	16.2	±	4.05 ^b^
PE 40:7	0.32	±	0.13 ^a^	0.41	±	0.11 ^b^	0.47	±	0.15 ^b^
PE P-18:1/22:6	0.89	±	0.24 ^a^	3.33	±	1.24 ^b^	3.67	±	1.07 ^b^
PE 40:8	0.15	±	0.04 ^a^	0.35	±	0.05 ^b^	0.43	±	0.09 ^c^
PE 42:7	0.02	±	0.00 ^a^	0.03	±	0.01 ^ab^	0.06	±	0.03 ^b^
PE 42:8	0.03	±	0.01 ^a^	0.05	±	0.03 ^ab^	0.08	±	0.03 ^b^
PE 44:6	0.06	±	0.04 ^a^	0.09	±	0.08 ^b^	0.10	±	0.08 ^b^

Different superscript letters in the same row indicate a significant group difference (*p* < 0.05).

**Table 9:** Concentrations (µmol/L) of plasma phosphatidylethanolamines (PE) of cats fed either a basic diet without or with fish oil at a supplementation dose of 0.5 or 1.0 g/kg body weight (BW)/day. Only PE that were decreased by the fish oil supplementation are displayed.

	Basic Diet			Fish Oil (0.5 g/kg BW/day)			Fish Oil (1.0 g/kg BW/day)		
	(*n* = 9)			(*n* = 10)			(*n* = 10)		
LPE 18:1	0.96	±	0.29 ^a^	0.52	±	0.06 ^b^	0.51	±	0.13 ^b^
LPE 18:2	1.62	±	0.60 ^a^	0.92	±	0.34 ^b^	0.81	±	0.30 ^b^
LPE 20:3	0.09	±	0.08 ^a^	0.03	±	0.03 ^b^	0.04	±	0.04 ^b^
LPE 20:4	0.76	±	0.20 ^a^	0.49	±	0.14 ^b^	0.41	±	0.13 ^b^
LPE 22:4	0.16	±	0.03 ^a^	0.06	±	0.03 ^b^	0.08	±	0.04 ^b^
LPE P-22:5	0.35	±	0.16 ^ac^	0.25	±	0.12 ^b^	0.31	±	0.15 ^c^
PE 33:1	0.19	±	0.03 ^a^	0.13	±	0.03 ^b^	0.12	±	0.03 ^b^
PE 33:2	0.17	±	0.03 ^a^	0.10	±	0.03 ^b^	0.10	±	0.02 ^b^
PE 34:1	0.25	±	0.08 ^a^	0.17	±	0.03 ^b^	0.16	±	0.03 ^b^
PE P-16:0/18:1	1.37	±	0.43 ^a^	1.01	±	0.25 ^b^	0.93	±	0.23 ^b^
PE 34:2	0.54	±	0.15 ^a^	0.37	±	0.11 ^b^	0.33	±	0.13 ^b^
PE P-16:0/18:2	1.23	±	0.34 ^a^	0.69	±	0.19 ^b^	0.63	±	0.15 ^b^
PE P-16:0/18:3	0.16	±	0.08 ^a^	0.07	±	0.03 ^b^	0.07	±	0.04 ^b^
PE 35:2	0.84	±	0.22 ^a^	0.64	±	0.22 ^b^	0.59	±	0.17 ^b^
PE 35:3	0.47	±	0.10 ^a^	0.31	±	0.10 ^b^	0.29	±	0.07 ^b^
PE 36:2	2.07	±	0.71 ^a^	1.15	±	0.23 ^b^	1.01	±	0.33 ^b^
PE P-18:0/18:2	4.70	±	1.02 ^a^	3.53	±	1.09 ^b^	3.12	±	1.03 ^b^
PE 36:3	0.60	±	0.18 ^a^	0.24	±	0.04 ^b^	0.22	±	0.05 ^b^
PE P-18:1/18:2	1.29	±	0.39 ^a^	0.81	±	0.23 ^b^	0.73	±	0.19 ^b^
PE 36:4	0.67	±	0.16 ^a^	0.45	±	0.09 ^b^	0.40	±	0.12 ^b^
PE 38:4	4.00	±	1.89 ^a^	2.50	±	0.62 ^b^	2.15	±	0.90 ^b^
PE P-16:0/22:4	2.52	±	0.74 ^a^	1.25	±	0.40 ^b^	1.12	±	0.41 ^b^
PE P-18:0/22:3	0.54	±	0.19 ^a^	0.36	±	0.20 ^b^	0.32	±	0.12 ^b^
PE P-18:0/22:4	3.08	±	0.78 ^a^	2.21	±	0.78 ^b^	1.90	±	0.58 ^b^

Different superscript letters in the same row indicate a significant group difference (*p* < 0.05).

**Table 10:** Concentrations (µmol/L) of plasma phosphatidylglycerols (PG) of cats fed either a basic diet without or with fish oil at a supplementation dose of 0.5 or 1.0 g/kg body weight (BW)/day.

	Basic Diet			Fish Oil (0.5 g/kg BW/day)			Fish Oil (1.0 g/kg BW/day)		
	(*n* = 9)			(*n* = 10)			(*n* = 10)		
PG 16:1_18:0	0.06	±	0.03 ^a^	0.13	±	0.03 ^b^	0.14	±	0.02 ^b^
PG 16:1_18:1	0.66	±	0.12 ^a^	0.50	±	0.06 ^b^	0.51	±	0.08 ^b^
PG 16:0_18:3	1.00	±	0.18 ^a^	0.74	±	0.15 ^b^	0.73	±	0.12 ^b^
PG 16:1_18:2	1.37	±	0.27 ^a^	0.91	±	0.18 ^b^	0.80	±	0.15 ^b^
PG 16:2_18:2	0.18	±	0.03 ^a^	0.13	±	0.05 ^b^	0.15	±	0.04 ^ab^
PG 18:1_18:1	0.78	±	0.08 ^a^	0.70	±	0.06 ^b^	0.69	±	0.07 ^b^
PG 18:0_18:3	1.24	±	0.23 ^a^	0.94	±	0.16 ^b^	0.85	±	0.15 ^b^
PG 18:1_18:2	3.14	±	0.53 ^a^	2.36	±	0.41 ^b^	2.17	±	0.29 ^b^
PG 16:0_20:4	0.21	±	0.02 ^a^	0.24	±	0.04 ^b^	0.27	±	0.03 ^c^
PG 18:1_18:3	0.22	±	0.04 ^a^	0.17	±	0.04 ^b^	0.17	±	0.04 ^b^
PG 18:2_18:2	0.56	±	0.14 ^a^	0.34	±	0.06 ^b^	0.33	±	0.08 ^b^
PG 16:1_20:4	0.15	±	0.06 ^a^	0.10	±	0.03 ^b^	0.10	±	0.03 ^b^
PG 18:2_18:3	0.18	±	0.07 ^a^	0.09	±	0.05 ^b^	0.08	±	0.03 ^b^
PG 16:0_22:1	0.33	±	0.05 ^a^	0.26	±	0.04 ^b^	0.26	±	0.05 ^b^
PG 18:2_20:0	0.22	±	0.08 ^a^	0.18	±	0.06 ^b^	0.17	±	0.05 ^b^
PG 18:1_20:3	0.76	±	0.12 ^a^	0.56	±	0.07 ^b^	0.56	±	0.10 ^b^
PG 18:1_20:4	2.42	±	0.53 ^a^	1.80	±	0.26 ^b^	1.80	±	0.27 ^b^
PG 18:2_20:3	1.39	±	0.32 ^a^	0.93	±	0.22 ^b^	0.84	±	0.18 ^b^
PG 18:1_20:5	0.32	±	0.08 ^a^	0.22	±	0.02 ^b^	0.23	±	0.04 ^b^
PG 18:2_20:4	4.82	±	1.11 ^a^	3.29	±	0.76 ^b^	2.85	±	0.47 ^b^
PG 18:2_20:5	0.29	±	0.09 ^a^	0.20	±	0.04 ^b^	0.19	±	0.06 ^b^
PG 18:0_22:1	0.47	±	0.09 ^a^	0.34	±	0.06 ^b^	0.31	±	0.03 ^b^
PG 18:1_22:1	0.23	±	0.06 ^a^	0.20	±	0.07 ^b^	0.22	±	0.05 ^ab^
PG 18:2_22:0	0.49	±	0.10 ^a^	0.35	±	0.06 ^b^	0.32	±	0.05 ^c^
PG 18:2_22:1	0.17	±	0.06 ^a^	0.10	±	0.05 ^b^	0.08	±	0.05 ^b^
PG 18:1_22:3	0.75	±	0.12 ^a^	0.62	±	0.09 ^b^	0.61	±	0.11 ^b^
PG 18:1_22:4	2.14	±	0.36 ^a^	1.74	±	0.22 ^b^	1.76	±	0.28 ^b^
PG 18:2_22:3	2.77	±	0.63 ^a^	1.96	±	0.40 ^b^	1.72	±	0.30 ^b^
PG 18:1_22:5	1.20	±	0.28 ^a^	0.83	±	0.18 ^b^	0.87	±	0.19 ^b^
PG 18:2_22:4	10.5	±	2.24 ^a^	7.40	±	1.36 ^b^	6.49	±	1.04 ^b^
PG 20:3_20:4	0.18	±	0.04 ^a^	0.15	±	0.04 ^b^	0.14	±	0.02 ^b^
PG 22:5_22:6	0.22	±	0.07 ^a^	0.34	±	0.10 ^b^	0.32	±	0.10 ^b^
PG 22:6_22:6	0.09	±	0.04 ^a^	0.42	±	0.10 ^b^	0.47	±	0.10 ^b^

Different superscript letters in the same row indicate a significant group difference (*p* < 0.05).

**Table 11:** Concentrations (µmol/L) of plasma phosphatidylinositols (PI), cholesterol esters (CE), phosphatidylserines (PS), diacylglycerols (DG), sphingomyelins (SM), fatty acids (FA), ceramides (CER), glycosylceramides, phosphatidic acids (PA), and further metabolites of cats fed either a basic diet without or with fish oil at a supplementation dose of 0.5 or 1.0 g/kg body weight (BW)/day.

	Basic Diet			Fish Oil (0.5 g/kg BW/day)			Fish Oil (1.0 g/kg BW/day)		
	(*n* = 9)			(*n* = 10)			(*n* = 10)		
Phosphatidylinositols									
PI 15:0_16:0	3.33	±	0.63 ^a^	2.43	±	0.47 ^b^	2.30	±	0.32 ^b^
PI 15:1_16:0	4.94	±	1.11 ^a^	3.94	±	0.83 ^b^	3.64	±	0.65 ^b^
PI 16:0_17:1	0.24	±	0.07 ^a^	0.29	±	0.05 ^b^	0.36	±	0.09 ^b^
PI 16:0_17:2	6.45	±	1.24 ^a^	4.81	±	0.65 ^b^	4.65	±	0.69 ^b^
PI 16:1_18:0	0.43	±	0.09 ^a^	0.48	±	0.09 ^ab^	0.52	±	0.09 ^b^
PI 16:1_18:1	1.68	±	0.38 ^a^	1.27	±	0.16 ^b^	1.33	±	0.21 ^b^
PI 16:1_18:2	4.04	±	0.89 ^a^	2.71	±	0.52 ^b^	2.48	±	0.39 ^b^
PI 17:1_18:1	11.2	±	2.09 ^a^	9.07	±	1.13 ^b^	8.84	±	1.29 ^b^
PI 17:1_18:2	48.2	±	10.3 ^a^	34.3	±	5.63 ^b^	29.1	±	4.18 ^c^
PI 16:0_20:0	0.54	±	0.20 ^a^	2.07	±	0.47 ^b^	2.35	±	0.53 ^b^
PI 18:0_18:0	0.40	±	0.09 ^a^	0.78	±	0.14 ^b^	0.83	±	0.20 ^b^
PI 16:0_20:3	0.42	±	0.07 ^a^	0.27	±	0.18 ^b^	0.33	±	0.12 ^ab^
PI 18:0_18:3	0.09	±	0.07 ^a^	0.19	±	0.10 ^b^	0.22	±	0.12 ^b^
PI 18:1_18:2	1.84	±	0.35 ^a^	1.31	±	0.33 ^b^	1.28	±	0.34 ^b^
PI 18:0_20:0	0.93	±	0.42 ^a^	3.93	±	0.76 ^b^	4.54	±	1.16 ^b^
PI 18:1_20:0	0.20	±	0.03 ^a^	0.41	±	0.10 ^b^	0.49	±	0.17 ^b^
PI 18:0_20:3	2.53	±	0.58 ^a^	2.36	±	0.63 ^ab^	2.21	±	0.50 ^b^
PI 18:1_20:3	0.30	±	0.12 ^a^	0.15	±	0.09 ^b^	0.10	±	0.05 ^b^
PI 18:1_20:4	1.82	±	0.45 ^a^	1.28	±	0.21 ^b^	1.31	±	0.31 ^b^
PI 18:1_20:5	0.09	±	0.00 ^a^	0.25	±	0.10 ^b^	0.36	±	0.10 ^c^
PI 18:2_22:6	0.26	±	0.05 ^a^	0.16	±	0.07 ^b^	0.15	±	0.07 ^b^
Cholesterol esters									
CE 14:0	1.74	±	0.51 ^a^	2.85	±	0.71 ^b^	4.08	±	1.23 ^c^
CE 15:0	2.43	±	0.88 ^a^	3.36	±	1.05 ^b^	4.48	±	1.21 ^c^
CE 16:0	93.0	±	48.8 ^a^	151	±	101 ^b^	185	±	130 ^c^
CE 16:1	18.9	±	6.75 ^a^	22.1	±	4.62 ^ab^	27.9	±	7.67 ^b^
CE 20:1	1.24	±	1.03 ^a^	2.28	±	1.03 ^b^	2.83	±	1.53 ^b^
CE 20:3	8.68	±	3.06 ^a^	6.64	±	0.94 ^b^	6.17	±	0.73 ^b^
CE 20:4	122	±	48.5 ^a^	146	±	33.9 ^b^	149	±	26.5 ^b^
CE 20:5	12.9	±	18.5 ^a^	486	±	149 ^b^	803	±	313 ^b^
CE 22:2	0.21	±	0.15 ^a^	0.35	±	0.16 ^b^	0.42	±	0.17 ^b^
CE 22:5	1.00	±	0.46 ^a^	1.52	±	0.33 ^b^	1.59	±	0.44 ^b^
CE 22:6	6.18	±	3.75 ^a^	35.8	±	8.43 ^b^	47.7	±	12.3 ^c^
Phosphatidylserines									
PS 36:4	0.35	±	0.08 ^a^	0.22	±	0.04 ^b^	0.23	±	0.04 ^b^
PS 36:5	0.60	±	0.12 ^a^	0.42	±	0.10 ^b^	0.37	±	0.06 ^b^
PS 38:4	0.24	±	0.05 ^a^	0.20	±	0.03 ^b^	0.19	±	0.04 ^b^
PS 38:5	1.16	±	0.23 ^a^	0.89	±	0.22 ^b^	0.81	±	0.13 ^b^
PS 38:6	0.44	±	0.08 ^a^	0.26	±	0.08 ^b^	0.23	±	0.04 ^b^
PS 38:7	0.48	±	0.10 ^a^	0.36	±	0.09 ^b^	0.30	±	0.07 ^c^
PS 40:5	0.01	±	0.00 ^a^	0.01	±	0.01 ^a^	0.02	±	0.03 ^b^
PS 40:8	0.22	±	0.03 ^a^	1.01	±	0.29 ^b^	1.38	±	0.40 ^c^
Diacylglycerols									
DG 16:1_18:1	0.78	±	0.49 ^a^	0.44	±	0.18 ^b^	0.70	±	0.47 ^ab^
DG 18:1_18:1	0.59	±	0.20 ^a^	0.38	±	0.11 ^b^	0.32	±	0.12 ^c^
DG 18:1_18:2	2.09	±	0.71 ^a^	1.62	±	0.29 ^b^	1.37	±	0.35 ^b^
DG 18:0_20:0	0.01	±	0.01 ^a^	0.01	±	0.00 ^a^	0.02	±	0.01 ^b^
DG 18:1_20:0	0.12	±	0.00 ^a^	0.19	±	0.10 ^ab^	0.40	±	0.39 ^b^
Sphingomyelins									
SM 40:4	0.11	±	0.07 ^a^	0.02	±	0.03 ^b^	0.01	±	0.00 ^b^
SM 41:2	4.61	±	1.07 ^a^	5.88	±	0.95 ^b^	6.60	±	1.11 ^c^
SM 42:1	15.7	±	3.20 ^a^	16.1	±	1.95 ^a^	18.7	±	3.13 ^b^
SM 42:2	14.1	±	2.84 ^a^	23.5	±	3.59 ^b^	29.1	±	4.67 ^c^
SM 44:2	0.38	±	0.15 ^a^	0.66	±	0.16 ^b^	0.73	±	0.23 ^b^
Fatty acids									
FA 20:1	2.44	±	0.77 ^a^	3.22	±	0.75 ^b^	3.38	±	0.72 ^b^
FA 20:2	1.57	±	0.39 ^a^	1.30	±	0.38 ^ab^	1.20	±	0.52 ^b^
FA 20:5	0.22	±	0.23 ^a^	5.73	±	1.98 ^b^	8.51	±	2.85 ^b^
FA 22:6	1.67	±	0.86 ^a^	7.07	±	1.60 ^b^	8.94	±	2.57 ^b^
Ceramides									
Cer d18:1/20:0	0.17	±	0.03 ^ac^	0.13	±	0.06 ^b^	0.17	±	0.04 ^c^
Cer d18:1/23:0	0.72	±	0.22 ^a^	0.60	±	0.08 ^b^	0.59	±	0.12 ^b^
Cer d18:1/24:1	0.35	±	0.09 ^a^	0.53	±	0.11 ^b^	0.58	±	0.17 ^b^
Glycosylceramides									
Hex2Cer d18:1/16:0	0.19	±	0.04 ^a^	0.21	±	0.05 ^b^	0.23	±	0.03 ^b^
Hex-Cer d18:1/18:0	0.25	±	0.09 ^a^	0.27	±	0.09 ^ab^	0.32	±	0.10 ^b^
Hex-Cer d18:1/24:1	0.85	±	0.19 ^a^	1.07	±	0.19 ^b^	1.27	±	0.26 ^c^
Phosphatidic acids									
PA 18:0_18:1	1.07	±	0.09 ^a^	0.75	±	0.31 ^b^	0.83	±	0.16 ^ab^
PA 18:0_18:2	0.69	±	0.15 ^ab^	0.51	±	0.19 ^b^	0.31	±	0.14 ^c^
PA 18:2_20:0	1.44	±	0.21 ^a^	0.99	±	0.26 ^b^	0.91	±	0.25 ^b^
Further metabolites									
Abscisic acid (AbsAcid)	0.01	±	0.00 ^a^	0.01	±	0.00 ^a^	0.01	±	0.00 ^b^
Acylcarnitine C0	22.1	±	4.58 ^a^	23.5	±	3.50 ^ab^	26.7	±	6.39 ^b^
Acylcarnitine C4	0.08	±	0.04 ^a^	0.10	±	0.02 ^b^	0.10	±	0.02 ^b^
Betaine	50.9	±	8.89 ^a^	44.4	±	8.48 ^b^	40.2	±	5.89 ^b^
Glutamine (Gln)	787	±	51.0 ^a^	898	±	154 ^b^	909	±	104 ^b^
Homoarginine (HArg)	1.34	±	0.52 ^a^	1.78	±	0.39 ^b^	1.75	±	0.64 ^b^
Hydroxyglutaric acid (OH-GlutAcid)	2.51	±	0.33 ^a^	2.27	±	0.17 ^b^	2.25	±	0.18 ^b^
Trigonelline	0.69	±	0.13 ^a^	0.61	±	0.10 ^b^	0.58	±	0.14 ^b^
Valine (Val)	170	±	31.8 ^a^	169	±	33.1 ^a^	148	±	27.1 ^b^

Different superscript letters in the same row indicate a significant group difference (*p* < 0.05).

## Data Availability

The raw data supporting the conclusions of this article will be made available by the authors upon request.

## Supplementary Materials

The following supporting information can be downloaded at: https://www.mdpi.com/article/10.3390/metabo16060427/s1, Supplementary Material S1: Plasma metabolites detected in this study.

## Abbreviations

The following abbreviations are used in this manuscript:

AbsAcid	Abscisic acid
BW	Body weight
CE	Cholesterol ester
CER	Ceramide
DHA	Docosahexaenoic acid
DG	Diacylglycerol
EPA	Eicosapentaenoic acid
FA	Fatty acid
FDR	False discovery rate
FIA MS/MS	Flow injection analysis-tandem mass spectrometry
Gln	Glutamine
HArg	Homoarginine
HEX-CER	Hexosylceramide
LC MS/MS	Liquid chromatography-tandem mass spectrometry
LPC	Lysophosphatidylcholine
LPE	Lysophosphatidylethanolamine
LPS	Lysophosphatidylserine
MCT	Medium-chain triacylglycerols
OH-GlutAcid	Hydroxyglutaric acid
PA	Phosphatidic acid
PC	Phosphatidylcholine
PC1	First principal component
PC2	Second principal component
PE	Phosphatidylethanolamine
PI	Phosphatidylinositol
PG	Phosphatidylglycerol
PS	Phosphatidylserine
SM	Sphingomyelin
TG	Triacylglycerol
Val	Valine
